# Opening a new route to multiport coherent XUV sources via intracavity high-order harmonic generation

**DOI:** 10.1038/s41377-020-00405-5

**Published:** 2020-09-24

**Authors:** Natsuki Kanda, Tomohiro Imahoko, Koji Yoshida, Akihiro Tanabashi, A. Amani Eilanlou, Yasuo Nabekawa, Tetsumi Sumiyoshi, Makoto Kuwata-Gonokami, Katsumi Midorikawa

**Affiliations:** 1 RIKEN Center for Advanced Photonics, RIKEN, Wako, Saitama 351-0198 Japan; 2 Photon Science Center, The University of Tokyo, Tokyo, 113-8656 Japan; 3 Intstitute for Solid State Physics, The University of Tokyo, Kashiwa, Chiba 277-8581 Japan; 4 Cyber Laser Inc., Wako, Saitama 351-0104 Japan; 5 Institute for Photon Science and Technology, The University of Tokyo, Tokyo, 113-0033 Japan; 6 Department of Physics, The University of Tokyo, Tokyo, 113-0033 Japan

**Keywords:** Ultrafast lasers, High-harmonic generation

## Abstract

High-order harmonic generation (HHG) is currently utilized for developing compact table-top radiation sources to provide highly coherent extreme ultraviolet (XUV) and soft X-ray pulses; however, the low repetition rate of fundamental lasers, which is typically in the multi-kHz range, restricts the area of application for such HHG-based radiation sources. Here, we demonstrate a novel method for realizing a MHz-repetition-rate coherent XUV light source by utilizing intracavity HHG in a mode-locked oscillator with an Yb:YAG thin disk laser medium and a 100-m-long ring cavity. We have successfully implemented HHG by introducing two different rare gases into two separate foci and picking up each HH beam. Owing to the two different HH beams generated from one cavity, this XUV light source will open a new route to performing a time-resolved measurement with an XUV-pump and XUV-probe scheme at a MHz-repetition rate with a femtosecond resolution.

## Extreme ultraviolet: generating coherent pulses inside a laser

A compact table-top laser system developed by researchers in Japan can generate extreme ultraviolet radiation (XUV) for ultrafast spectroscopy. Coherent XUV pulses are used to accurately measure data on electronic transitions in atoms and molecules, however they can usually only be generated via synchrotron radiation from large particle accelerators. A possible route to generating XUV on a smaller lab scale is to direct high-order harmonic pulses from a femtosecond laser onto a gas. Katsumi Midorikawa at the RIKEN Center for Advanced Photonics in Wako and co-workers have developed a novel method where the gas targets are actually placed inside the laser cavity. By using two different gases, they can simultaneously generate an XUV pump and a probe, opening a route to very accurate timing of atomic measurements, perhaps resolving events as short as a few femtoseconds.

## Introduction

Synchrotron radiation (SR) and an X-ray free electron laser (XFEL), which are based on large-scale particle accelerators, are the current standards for XUV~ X-ray light sources in multiuser facilities^1^. A high degree of spatial coherence and a high repetition rate of more than 100 MHz for SR are beneficial for acquiring accurate spectroscopic data of materials; however, the temporal characteristic of SR is not adequate for resolving the matter dynamics in the less-than-picosecond regime because of the low temporal coherence and relatively long pulse duration of 10 ps or longer. An XFEL, in contrast, can generate an ultrashort X-ray pulse with a duration of less than 10-40 fs. The intensity of the pulse delivered from an XFEL can be sufficiently high to cause a nonlinear interaction with matter, which paves the way for the investigation of novel phenomena in high-intensity physics. Nevertheless, the relatively low repetition frequency of XFEL pulses, typically in the range of 10–100 Hz, is an obstacle to the execution of pump and probe measurements, with repeated delay scans requiring a long machine time in a huge facility.

The high-order harmonic (HH) pulse of an intense femtosecond laser generated upon its interaction with a gas medium has been expected to be another coherent XUV light source over the last two decades. This is because the HH pulse is as highly coherent as the fundamental laser pulse in both the spatial and temporal domains, and the pulse duration can be shortened to a few femtoseconds or even the attosecond regime^2^. The laser system that delivers the fundamental femtosecond laser pulses can be situated on an optical table in a conventional university laboratory space such that researchers can exclusively use coherent XUV HH pulses whenever they want.

However, the repetition rate of the HH pulse, limited by the repetition rate of the fundamental laser system^3^, is still insufficient for novel applications, such as ultrafast XUV spectroscopy^4^, photoelectron spectroscopy^5^, and coincidence measurements using a velocity map imaging detector^6^ or a reaction microscope^7^. In particular, an exceedingly high photon flux is necessary for implementing time-resolved high-precision photoemission spectroscopy, while the pulse energy should be lower than the threshold to induce the space charge effect, which distorts the photoelectron spectrum. Therefore, a repetition rate higher than the MHz order is mandatory. In addition, the bandwidth of the XUV pulse (Δ*E*) should be narrower than 10^-2^ × the photon energy (*E*) for high-resolution spectroscopy^8^, although the pulse duration should be kept in the femtosecond regime to resolve ultrafast dynamics.

To meet these requirements, challenges to improve high-order harmonic generation (HHG) driving laser systems have gradually been established with recent advances in solid-state laser technologies^9–14^. A fibre laser amplifier system, for example, was developed to generate HH pulses with a repetition rate of 1 MHz owing to efficient heat removal from a 100 W class rod-type fibre amplifier. Although the low damage threshold of a fibre amplifier with a small mode field diameter had been considered to be a drawback that limits the amplified pulse energy to approximately 100 μJ, a fibre laser system delivering 1 mJ pulses with a repetition rate of 0.1 MHz was realized with the state-of-the-art technique adopting the coherent combination of output beams from multiple large-pitch fibres^12^. We note, however, that the average power of this type of fibre laser system has plateaued around the 100 W regime; hence, we still need to achieve ‘1 mJ and 1 MHz’ pulses, requiring an average power of 1 kW.

The concept of coherent combination of multiple pulses can be turned into another concept of an enhancement cavity to temporally accumulate many separated pulses provided by a mode-locked oscillator or an amplifier with a 100 MHz class repetition rate^15–20^. This concept is reasonable for HHG sources. Intense fundamental laser pulses delivered from a laser amplifier system are usually dumped after HHG, whereas fundamental pulses are repeatedly used for HHG in an enhancement cavity, resulting in an average power of more than 1 kW. Since the first demonstration of HHG in an enhancement cavity, the generated HH pulses have been utilized as a frequency comb in the vacuum ultraviolet (VUV) or XUV wavelength region to accurately measure the spectroscopic data of atomic transitions. One main drawback of this light source, from the viewpoint of users, is that the system is rather complicated and somewhat fragile with respect to external perturbations. The system reported in ref. ^18^ consists of four parts: a mode-locked fibre oscillator, a fibre amplifier chain, a nonlinear pulse compressor, and an enhancement cavity. In addition, the repetition frequency of the oscillator must be kept at the circulating frequency of the pulse in the enhancement cavity with subwavelength precision by using servo loop electronics, although the nonlinear phase shift induced in the HHG process modulates the circulating frequency.

To achieve robustness for pulse accumulation in a cavity, we proposed and demonstrated a new method of realizing MHz HHG^21^, by which HH pulses are generated in the cavity of a high-power femtosecond oscillator. This approach is more advantageous than the existing enhancement cavity methods because there is no need to stabilize the repetition frequency of the oscillator and thus no need to compensate for the frequency modulation induced by the nonlinear phase shift in the HHG process. The latter characteristic is very important because we can design the oscillator cavity to include multiple focusing points to separately generate HH pulses without accurate estimation of the nonlinear phase shifts depending on the laser pulse intensity and the gas target condition.

## Results

### High-pulse-energy mode-locked oscillator

**Fig. 1:**
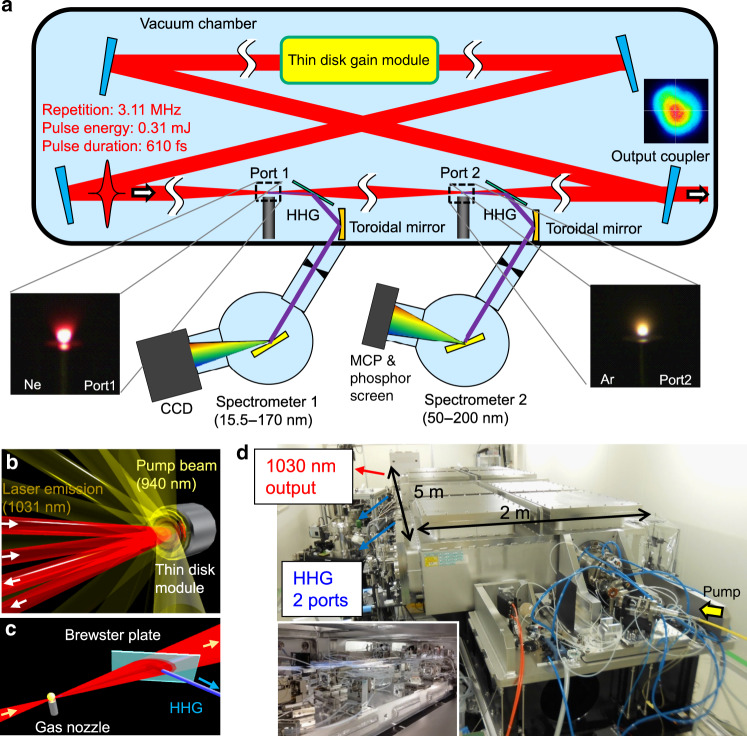
Setup for multiport coherent XUV source. **a** Schematic of the mode-locked oscillator developed in this work. The laser is configured as a ring cavity in which a mode-locked pulse unidirectionally circulates. The cavity length is approximately 100m, and the resultant pulse circulation frequency is 3.11MHz. An Yb:YAG thin disk is adopted as the laser gain medium. We insert two sets of telescopes composed of two concave mirrors in the ring cavity to tightly focus the circulating mode-locked pulse at separate points. A gas nozzle at each focusing point independently delivers the gas target to generate HH pulses. The generated HH pulse from each focusing point is split from the ring cavity with a Brewster plate made of sapphire and then introduced into a spectrometer to resolve the spectrum of the HH pulse. A picture of the plasma luminescence from the Ne gas target injected to a focusing point is shown on the bottom left, and that from the Ar gas target injected to another focusing point at the same time is shown on the bottom right. **b** Schematic view of the gain module in laser operation. The module consists of an Yb:YAG thin disk attached to a diamond heat sink and a retroreflector system to arrange 24 bounces of the pumping beam on the thin disk. The double reflections of the mode-locked pulse from the thin disk in one circulation in the ring cavity ensure a laser gain equivalent to that in a linear cavity. **c** Schematic view of the Brewster plate reflecting HH pulses. **d** Picture of the laser system. The entire cavity is enclosed in a vacuum chamber. Some of the devices and instruments in the vacuum chamber with bundles of tubes circulating cooling water are shown in the inset

To demonstrate intracavity HHG, we have developed a novel mode-locked oscillator distinct from the oscillator reported in^21^. A schematic of the oscillator cavity is shown in Fig. 1(a). We use an Yb:YAG thin disk as a gain medium^22–25^ because it is suitable for high-average-power oscillation with efficient heat removal from the back side of a heat sink attached to the thin disk^26^, and we have already ensured the stable Kerr lens mode locking of an Yb:YAG thin disk oscillator with an intracavity average power nearly reaching 1 kW^21^. The configuration of the beam paths related to the thin disk module is depicted in Fig. 1b.

The cavity is designed for one-way HHG at a tight focusing point and is configured as a ring resonator. A continuous flow of a gas target is supplied via a cylindrical nozzle placed near the focus. The generated HH pulses are decoupled from the cavity with a Brewster plate made of sapphire with a thickness of 1 mm, as shown in Fig. 1c. We call this setup including a telescope, a gas target, and a Brewster plate for the HHs an HHG port. The reflected harmonic pulses are introduced into an XUV or VUV spectrograph by reflection off a gold-coated toroidal mirror situated such that the HH pulse is focused into the entrance slit of the spectrometer.

**Fig. 2:**
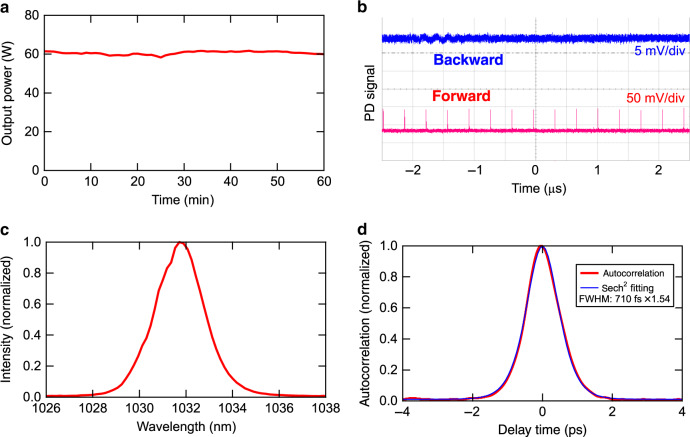
Output properties of high-energy mode-locked oscillator. **a** History of the average power of the output pulse from the mode-locked oscillator. **b** Bottom trace: signal from a PIN photodiode detecting the beam circulating in the forward direction. Top trace: signal from a PIN photodiode detecting the beam circulating in the backward direction. **c** Typical spectrum of a mode-locked pulse. **d** Autocorrelation trace of the output pulse from the mode-locked oscillator depicted as a red curve. A fit assuming a sech^2^ pulse shape is also shown as a blue curve

The length of the cavity is adjusted to 100 m such that the repetition rate of the mode-locked pulses should be approximately 3 MHz, where cumulative ionization effects in gas targets can be avoided. The optical table is water-cooled, and the entire cavity on the optical table is set in a vacuum chamber to eliminate nonlinear phase shifts and index fluctuations with the flow of air during the 100 m propagation of the pulse, as shown in Fig. 1d. Other details of the cavity are described in the “Methods” and Supplementary Information. We show the time evolution of the output power of the mode-locked pulses from the oscillator in Fig. 2a at a pumping laser diode power of 1.22 kW. The standard deviation of the fluctuation of the output power is only 1.3% of the average power of 60.6 W. Unidirectional mode locking was achieved, as confirmed by observing the pulse leakage in the forward and backward directions from a high-reflectivity mirror used in the cavity. The signal detected with a fast PIN photodiode in each direction is shown in Fig. 2b. There is no notable pulse in the backward direction, while a pulse train with a repetition rate of 2.87 MHz and an approximately 350 ns pulse interval appears in the forward direction without visible subpulses. We estimate the pulse energy in the cavity to be 0.70 mJ by considering a reflectivity of 97% for an output coupler, the measured output average power, and a repetition rate of 2.87 MHz.

The spectrum of the output pulse exhibits a peak at 1032 nm with a spectral width of 2.3 nm, as shown in Fig. 2c. The pulse duration is estimated to be 710 fs from the measured autocorrelation trace shown in Fig. 2d by assuming the sech^2^ pulse shape. The pulse duration is consistent with that obtained from the measured spectrum at the Fourier limit. We conclude from the measured pulse duration and pulse energy that the peak power of the intracavity pulse reaches 0.87 GW. This is equivalent to ten-fold the magnitude of the highest intracavity average power ever obtained from a mode-locked oscillator developed for intracavity HHG^3,17,25^.

### Single-port intracavity HHG

**Fig. 3:**
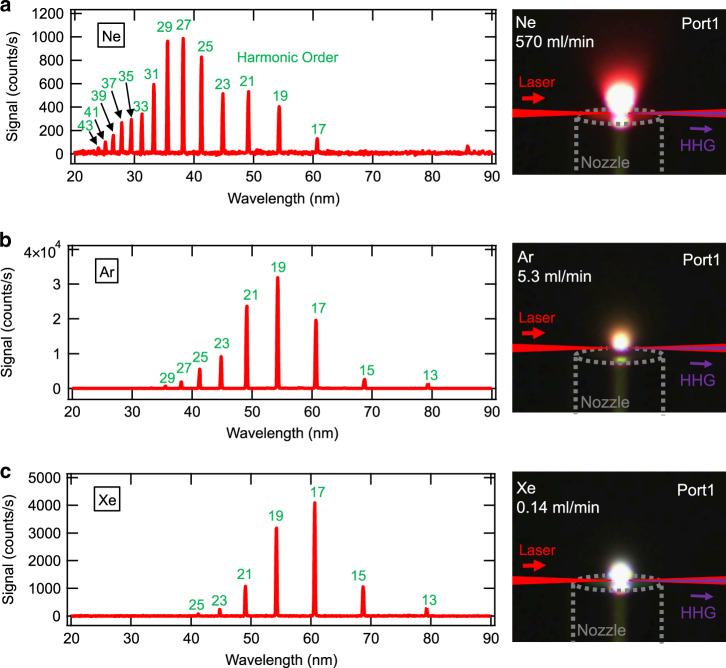
HH spectra obtained under single-HHG-port operation of the mode-locked oscillator. **a** HH spectrum generated from the Ne gas target. **b** HH spectrum generated from the Ar gas target. **c** HH spectrum generated from the Xe gas target. An image of the plasma luminescence for each gas target is exhibited to the right of each HH spectrum figure. The nozzle supplying the gas target is shown as grey dashed lines, and the propagation axes of the laser and HH pulses are schematically shown as red and purple lines, respectively, in each image

In our first HHG experiment, we examined the performance of a single-HHG port with a repetition rate of 3.11 MHz by supplying Ne, Ar, and Xe gases as the HHG targets. We measured HH spectra from the gas targets Ne, Ar, and Xe using an XUV grazing incidence spectrograph, as shown in Fig. 3a–c, respectively. We injected the Ne, Ar, and Xe gases at flow rates of 570 ml/min, 5.3 ml/min, and 0.14 ml/min, respectively, for these measurements. Note that the energy of the fundamental pulse was reduced to 0.31 mJ in this HHG examination because sudden injection of a gas target might induce Q-switch mode locking that intermittently generates giant pulses, which possibly cause damage to the optics in the cavity under the maximum pulse energy condition. The pulse duration was also reduced to 610 fs, which is inherent to a low pulse energy. We estimated the peak intensity of the pulse at the focusing point of the HHG port to be 8.3×10^13^ W/cm^2^ by considering the beam diameter of 37 μm obtained from the cavity design described in the Supplementary Information. The mode locking stability was not significantly changed by the injection and interruption of each gas target.

The shortest cutoff wavelength in the HH spectrum shown in Fig. 3a was 24 nm, which was equivalent to the cutoff photon energy $\hbar \omega _c$ of 52 eV and the harmonic order of 43. The cutoff photon energy should be related to the ponderomotive energy of the fundamental laser pulse *U*_*p*_ and the ionization energy *I*_*p*_, as shown by $\hbar \omega _c = I_p + 3.17 \times U_p$, according to the three-step model of HHG^27^. The cutoff photon energy was determined to be 48 eV from the estimated intensity of the fundamental pulse and the ionization energy of the Ne atom. Thus, the measured $\hbar \omega _c$ in Fig. 3a agrees well with the $\hbar \omega _c$ estimated from the intensity of the fundamental laser pulse. The cutoff photon energies of HH pulses from Ar and Xe were determined to be 40 eV and 32 eV, respectively, from the measured spectra shown in Figs. S5b and S5c in the Supplementary Information with a logarithmically scaled intensity. These are relatively low compared to those expected from the three-step model, 42 eV and 38 eV, respectively. We consider that a high degree of ionization might suppress HHG in both gas targets owing to the high intensity of the fundamental pulse. The photon energy bandwidth to peak photon energy ratio, Δ*E*/*E*, was at most 8×10^−3^. The maximum photon flux of H17 was obtained by adjusting the gas target flow, resulting in 1.4×10^10^ photons/s, which is equivalent to an average power of 47 nW. The details of the measurements for Δ*E*/*E* and the photon flux are described in the Supplementary Information.

The conversion efficiency from the fundamental laser pulse in the cavity with an average power of 0.96 kW to the H17 pulse was estimated to be 4.9×10^−11^, which was lower than that expected from the conventional HHG process^28^. This might be due to the high degree of ionization of the gas target medium, which caused depletion of neutral atoms as HHG sources and degraded the phase-matching condition between the fundamental and HH pulses. We expect that the conversion efficiency will be improved by shortening the pulse duration to suppress the ionization.

### Multiport intracavity HHG

**Fig. 4:**
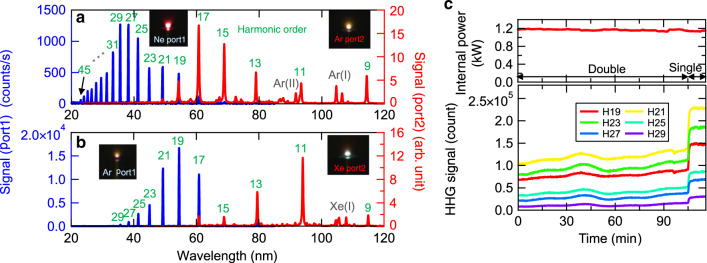
HH spectra obtained under double-HHG-port operation of the mode-locked oscillator. **a** HH spectrum generated from the Ne gas target injected into the first HHG port depicted as a blue curve, and that generated from the Ar gas target injected into the second HHG port depicted as a red curve. **b** HH spectrum generated from the Ar gas target injected into the first HHG port depicted as a blue curve, and that generated from the Xe gas target injected into the second HHG port depicted as a red curve. **c** History of the intracavity average power of the mode-locked oscillator (top panel) and that of the harmonic signal at each HH order obtained from the first HHG port (bottom panel) under double-HHG-port operation

Next, we added another HHG port into the cavity. The injection of the Ne gas target into the first HHG port and its interruption and those for the Ar gas target at the second HHG port did not discontinue the mode locking of the laser even when both gas targets were sequentially injected or interrupted. We show the plasma luminescence from the Ne gas target in the bottom left picture and that from the Ar gas target in the bottom right picture in Fig. 1a. Both gas targets were simultaneously injected during these observations. The spectra of the HH pulses from the first (Ne gas target) and second (Ar gas target) HHG ports are depicted in Fig. 4a as blue and red curves, respectively. The former HH spectrum is very similar to that obtained from the Ne gas target of the single-HHG port in the cavity previously mentioned [Fig. 3a]; thus, we conclude that the temporal characteristic of the fundamental pulse was not significantly changed by the operation of the double-HHG ports using different gas targets. In contrast, we observe that the cutoff photon energy of the HH spectrum from Ar in Fig. 4a is lower than that of the HH spectrum in Fig. 3b despite using the same Ar gas for injection. This is simply due to the significant reduction in the diffraction efficiency of the Al-MgF_2_-coated diffraction grating in the VUV normal incidence spectrograph (VTM300, HORIBA JOVIN YVON) used in the second HHG port. On the other hand, the diffraction efficiency of the grating (No. 54100 210, HORIBA JOVIN YVON) in the XUV grazing incidence spectrograph attached to the first HHG port is optimized for a wavelength of ~30 nm. Another feature, the appearance of line emissions from Ar atoms and Ar ions on the red curve in Fig. 4a, originates from the high correction efficiency for the incoherent 4π steradian emission from the second HHG port. This is because the diameter of the aperture used for the second HHG port is larger than that of the aperture used for the first HHG port. The high sensitivity of the microchannel plate (MCP) employed for detecting the HH spectrum from the second HHG port also contributes to the increase in spectral intensity at wavelengths longer than 60 nm, where the X-ray CCD camera used to observe the HH spectrum from the first HHG port is considerably insensitive owing to the low transmission of light at these wavelengths in silicon. The line emission from Ar ions supports our supposition regarding the suppression of the HH yield with a high degree of ionization. We observed that the spatial profile of each HH pulse at the entrance slit of the VUV spectrograph was Gaussian like, whereas the spatial profiles of the line emissions were partially distorted.

We demonstrated another double-port HHG by supplying Ar gas to the first HHG port and Xe gas to the second HHG port. We were able to measure the HH spectra generated from Ar and Xe simultaneously, shown as blue and red curves in Fig. 4b, respectively, without readjustment of the laser cavity.

We investigated the stabilities of the fundamental and HH pulses for more than one hour. The time evolution of the average power of the fundamental pulse in the cavity and that of the HH signal at each order are shown in the top and bottom panels of Fig. 4c, respectively. We simultaneously conducted double-port HHG by injecting Ar gas targets into the nozzles of both the first and second ports. We recorded the HH spectra from the first HHG port with the XUV grazing incidence spectrograph. Each HH spectrum at each order was integrated around each peak, resulting in an HH signal at each order as shown in Fig. 4c.

The rms error of the HH signal at H21, indicating the instability of the pulse energy of H21, is estimated to be 6.0% before the sudden increase in the intensity of the signal at 105 min., whereas that of the intracavity average power is 1.2%, indicating that the stability of the HH pulse was degraded compared with the stability of the fundamental pulse. This is reasonable because the HHG is a highly nonlinear process. We consider that the power instability of the fundamental laser pulse originated from the power and wavelength fluctuations of the laser diode pumping the thin disk laser. A high stability of the temperature of the cooling water for the laser diode is required to improve the fundamental laser stability.

We note that the concurrent increases in the intensities of all the harmonic signals in Fig. 4c at 105 min. were caused by our intentional interruption of the injection of the Ar gas target into the nozzle in the second HHG port. It was confirmed from this examination that the mode locking was robust against a sudden change in the gas target injection condition. The increase in the intensity of each HH signal delivered from the first working HHG port was attributed to a decrease in the ambient Ar gas pressure, which acted as an absorber of the generated HH pulses.

## Discussion

We demonstrated intracavity HHG using a mode-locked ring oscillator, which adopted an Yb:YAG thin disk as a gain medium, with a repetition rate of 3.11 MHz and a pulse energy of 0.31 mJ. Double-HHG ports were successfully built in the cavity and could be independently operated with different gas targets without disturbing the mode locking. Continuous HHG from the double-HHG ports was implemented for more than one hour. We took a step closer to the practical use of MHz HH pulses as a coherent XUV light source, as indicated by these experimental results, although we know that further improvement of the performance is necessary by comparing the HH light source performance with that of other existing state-of-the-art HH light sources, as shown in Table 1.

**Table 1:** List of the key performance metrics of this work and related state-of-the-art works

Ref.	Laser source	HHG method	Pulse energy (μJ)	Pulse duration	Peak intensity (10^13^ W/cm^2^)	Repetition rate	Harmonic order range	XUV average power	ΔE/E
this work	Yb:YAG Thin disk oscillator	Intracavity HHG	310	610 fs	8.3	3.11 MHz	H19-45 & H9-17 (dual)	47 nW @H17	8 × 10^−3^
25	Yb:YAG Thin disk oscillator	Intracavity HHG	18	255 fs	2.8	17.35 MHz	H11-17	0.55 nW @H11	5.6 × 10^−2^
9	Yb fibre CPA	single pass	28	267 fs	2	1 MHz	<H15	870 nW @H15	2.1 × 10^−2^
10	Yb fibre CPA + Post compression	single pass	7.1	31 fs	7.4	10.7 MHz	H23	51.1 μW @H23	1.7 × 10^-2^
11	Yb fibre CPA + Post compression	single pass	90 (SH)	85 fs	21	120 kHz	H9-13 of SH	832 μW @H9 of SH	1.8 × 10^−3^
12	Yb fibre CPA + Post compression	single pass	500	35 fs	>20	100 kHz	H47-59	2.2 μW @H57	1.9 × 10^−2^
13	TD Oscillator + Post compression	single pass	19	108 fs	5.5	2.4 MHz	H15-25	0.18 nW @H19	1.2 × 10^−2^
17	Yb fibre comb	Enhancement cavity	52	120 fs	9	154 MHz	H9-27	22 μW @ H11	3.0 × 10^−2^
18	Yb fibre	Enhancement cavity	30	175 fs or 57 fs	8	78 MHz	<H95	5.3 μW @H27	8.2 × 10^−3^
19	Yb fibre	Enhancement cavity	100	160 fs	3.6	10 MHz	H7-35	500 μW @H7	3.6 × 10^−3^
20	Yb fibre	Enhancement cavity	170	120 fs	21	60 MHz	H13-35	520 nW @H27	<5.5 × 10^−4^

The average power of the HH pulse of our HH light source appears to be low although the pulse energy is significantly high and comparable to that used for single pass HHG. This low conversion efficiency is due to the long pulse duration of the mode-locked oscillator, which causes a high degree of ionization of the gas target before reaching the pulse peak, which in turn degrades the efficiency of conversion to the HH pulse. Pulse shortening is expected by using other Yb-doped laser materials, such as Yb:Lu_2_O_3_^29^ and Yb:CALGO^30^, with broader gain spectral profiles. Heat generation by the unused pump power of the laser diode significantly deforms the thin disk and limits the highest average power of laser oscillation. Efficient pump power transfer to an Yb gain material should be realized by tuning the wavelength of the pumping laser diode to the zero-phonon absorption line of the gain material^31^, alleviating the thermal issue in laser oscillation. The accumulation of intracavity pulses will be promoted and the pumping laser power will be reduced by increasing the finesse of the laser cavity. An upgraded MHz XUV light source based on the HH pulses will be realized after resolving these issues.

## Material and methods

We adopted an Yb:YAG thin disk attached to a diamond heat sink (Dausinger+Giesen GmbH, TD-25-7-wedge-diamond) as a gain medium in a mode-locked ring oscillator for intracavity HHG. The diameter of the disk was 25 mm, the thickness was 250 μm, and the Yb doping concentration was 7 mol%. The disk surfaces were polished at a wedge angle of 0.05 degrees. The pumping light for the thin disk was delivered from a laser diode through a multimode fibre with a wavelength of 940 nm (Laserline GmbH, LDF13000), and the output beam from the fibre was collimated with a lens and irradiated on the disk 24 times with a retroreflector module. The collimation of the lens was slightly defocused such that the pump beam size should be approximately 10 mm in full width at half maximum (FWHM) accompanied by a blurred edge to relax the heat strain at the sharp edge of the pump beam with a top-hat profile generated by the optimally adjusted collimation lens, resulting in a pump beam diameter of approximately 15 mm in full width at 25% maximum.

The mode-locked oscillator was configured as a ring cavity, and the cavity length was designed to be approximately 100 m for a pulse repetition rate of 3 MHz, as described in the main text. All the optics involved in the cavity were fixed in water-cooled holders to avoid thermal deformation or damage caused by the heat of the kW-level intracavity average power in the vacuum environment. The radius of curvature of the concave Yb:YAG thin disk in the cavity was 3.7 m without pumping, while it increased with the thermal deformation induced by the irradiation of pump light from the laser diode. We estimated this thermal lens effect with the assumption that the inverse of the curvature linearly decreases with increasing pump laser power. As a result, we put a concave mirror, which is referred to as, thermal lens compensation concave mirror (TLCCM)’ in the Supplementary Information, next to the thin disk. We were able to compensate for the change in the thin disk curvature by adjusting the separation between the thin disk and the TLCCM to keep the laser oscillation of the cavity stable. Note that the beam path was folded with the thin disk two times to acquire a sufficient gain from the thin disk. We initiated the mode locking by shaking a motorized linear translation stage fixing two pairs of folding mirrors placed between the thin disk and the TLCCM.

We employed the Kerr lens mode locking scheme to obtain femtosecond pulses on the basis of the successful Kerr lens mode locking demonstrated in a previous work for the high-average-power ring oscillator with a cavity length of approximately 20 m^21^. Therefore, we utilized a wedged YAG plate with a thickness of 6 mm and with antireflection coatings on both surfaces as the Kerr lens medium. We also used a hard aperture to induce gain modulation via the Kerr lens effect. The position of the YAG plate and that of the hard aperture in the ring cavity were carefully adjusted to achieve unidirectional mode locking^32^. Other details of the cavity design are described in the Supplementary Information.

We also designed a linear cavity and performed a preliminary experiment for Kerr lens mode locking before developing the ring oscillator because the design of the linear cavity without considering the propagation direction is much easier than that of the ring cavity. We achieved an intracavity pulse energy of more than 1 mJ in this experiment. The performance of the linear mode-locked oscillator in the preliminary experiment is described in the Supplementary Information.

Gires–Tournois interferometer (GTI) mirrors were installed to give a negative group delay dispersion (GDD) to a femtosecond pulse circulating in the ring cavity to compensate for the positive GDD and nonlinear phase shift accumulated during the propagation in transmitting materials. The total GDD provided by the GTI mirrors was evaluated to be −23,000 fs^2^.

## Supplementary information

Supplementary Information for Opening a new route to multiport coherent XUV sources via intracavity high-order harmonic generation

## References

[1] Bilderback DH, Elleaume P, Weckert E (2005). Review of third and next generation synchrotron light sources. J. Phys. B: . Mol. Opt. Phys..

[2] Krausz F, Ivanov M (2009). Attosecond physics. Rev. Mod. Phys..

[3] Labaye F (2019). XUV sources based on intra-oscillator high harmonic generation with thin-disk lasers: current status and prospects. IEEE J. Sel. Top. Quantum Electron..

[4] Bressler C, Chergui M (2004). Ultrafast x-ray absorption spectroscopy. Chem. Rev..

[5] Schultze M (2010). Delay in photoemission. Science.

[6] Kling MF, Vrakking MJJ (2008). Attosecond electron dynamics. Annu. Rev. Phys. Chem..

[7] Ullrich J (2003). H. Recoil-ion and electron momentum spectroscopy: reaction-microscopes. Rep. Prog. Phys..

[8] Hellmann S, Rossnagel K, Marczynski-Bühlow M, Kipp L (2009). Vacuum space-charge effects in solid-state photoemission. Phys. Rev. B.

[9] Boullet J (2009). High-order harmonic generation at a megahertz-level repetition rate directly driven by an ytterbium-doped-fiber chirped-pulse amplification system. Opt. Lett..

[10] Hädrich S (2015). Exploring new avenues in high repetition rate table-top coherent extreme ultraviolet sources. Light.: Sci. Appl.

[11] Klas R (2016). Table-top milliwatt-class extreme ultraviolet high harmonic light source. Optica.

[12] Rothhardt J (2016). High-repetition-rate and high-photon-flux 70 eV high-harmonic source for coincidence ion imaging of gas-phase molecules. Opt. Express.

[13] Emaury F, Diebold A, Saraceno CJ, Keller U (2015). Compact extreme ultraviolet source at megahertz pulse repetition rate with a low-noise ultrafast thin-disk laser oscillator. Optica.

[14] Nagy T (2019). Generation of three-cycle multi-millijoule laser pulses at 318 W average power. Optica.

[15] Jones RJ, Moll KD, Thorpe MJ, Ye J (2005). Phase-coherent frequency combs in the vacuum ultraviolet via high-harmonic generation inside a femtosecond enhancement cavity. Phys. Rev. Lett..

[16] Gohle C (2005). A frequency comb in the extreme ultraviolet. Nature.

[17] Cingöz A (2012). Direct frequency comb spectroscopy in the extreme ultraviolet. Nature.

[18] Pupeza I (2013). Compact high-repetition-rate source of coherent 100 eV radiation. Nat. Photon.

[19] Ozawa A, Zhao Z, Kuwata-Gonokami M, Kobayashi Y (2015). High average power coherent vuv generation at 10 MHz repetition frequency by intracavity high harmonic generation. Opt. Express.

[20] Mills AK (2019). Cavity-enhanced high harmonic generation for extreme ultraviolet time- and angle-resolved photoemission spectroscopy. Rev. Sci. Instrum..

[21] Eilanlou AA, Nabekawa Y, Kuwata-Gonokami M, Midorikawa K (2014). Femtosecond laser pulses in a Kerr lens mode-locked thin-disk ring oscillator with an intracavity peak power beyond 100 MW. Jpn. J. Appl. Phys..

[22] Pronin O (2011). High-power 200 fs Kerr-lens mode-locked Yb:YAG thin-disk oscillator. Opt. Lett..

[23] Saraceno CJ (2013). Cutting-edge high-power ultrafast thin disk oscillators. Appl. Sci..

[24] Brons J (2014). Energy scaling of Kerr-lens mode-locked thin-disk oscillators. Opt. Lett..

[25] Labaye F (2017). Extreme ultraviolet light source at a megahertz repetition rate based on high-harmonic generation inside a mode-locked thin-disk laser oscillator. Opt. Lett..

[26] Giesen A (1994). Scalable concept for diode-pumped high-power solid-state lasers. Appl. Phys. B..

[27] Corkum PB (1993). Plasma perspective on strong-field multiphoton ionization. Phys. Rev. Lett..

[28] Takahashhi EJ (2002). Generation of highly coherent submicrojoule soft x rays by high-order harmonics. Phys. Rev. A..

[29] Endo M, Ozawa A, Kobayashi Y (2013). 6-GHz, Kerr-lens mode-locked Yb:Lu_2_O_3_ ceramic laser for comb-resolved broadband spectroscopy. Opt. Lett..

[30] Klenner A, Golling M, Keller U (2014). High peak power gigahertz Yb:CALGO laser. Opt. Express.

[31] Weichelt B, Voss A, Ahmed MA, Graf T (2012). Enhanced performance of thin-disk lasers by pumping into the zero-phonon line. Opt. Lett..

[32] Heatley DR, Dunlop AM, Firth WJ (1993). Kerr lens effect in a ring resonator with aperture: mode locking and unidirectional operation. Opt. Lett..

