# Supplementary material for: Opening a new route to multiport coherent XUV sources via intracavity high-order harmonic generation
Source: Light Sci Appl. 2020 Sep 24;9:168. doi: 10.1038/s41377-020-00405-5 (PMC7511353; doi:10.1038/s41377-020-00405-5)
Supplement: Supplementary file 1 — Supplementary Information for Opening a new route to multiport coherent XUV sources via intracavity high-order harmonic generation [file 41377_2020_405_MOESM1_ESM.docx]

***Supplementary Information for***

**Opening a new route to multiport coherent XUV sources via intracavity high-order harmonic generation**

Natsuki Kanda^1,2,3^, Tomohiro Imahoko^4^, Koji Yoshida^4^, Akihiro Tanabashi^1^,

A. Amani Eilanlou^1^, Yasuo Nabekawa^1^, Tetsumi Sumiyoshi^4^, Makoto Kuwata-Gonokami^5,6^, and Katsumi Midorikawa^1,5*^

*^1^RIKEN Center for Advanced Photonics, RIKEN, Wako, Saitama 351-0198, Japan*

*^2^Photon Science Center, The University of Tokyo, Tokyo 113-8656, Japan*

*^3^Intstitute for Solid State Physics, The University of Tokyo, Kashiwa, Chiba 277-8581, Japan*

*^4^Cyber Laser Inc., Wako, Saitama 351-0104, Japan*

*^5^Institute for Photon Science and Technology, The University of Tokyo, Tokyo 113-0033, Japan*

*^6^Department of Physics, The University of Tokyo, Tokyo 113-0033, Japan*

^*^*Corresponding author: kmidori@riken.jp*

**CAVITY DESIGN AND PERFORMANCE**

**A. Demonstration of linear cavity**

Before designing and building the ring cavity of a mode-locked oscillator, we demonstrated the mode-locking laser oscillation in a linear cavity with a length of 50 m to confirm the feasibility of the high-energy mode-locked oscillator under the situation realized much easier than a ring cavity. We simulated a laser beam propagation in the linear cavity using the ABCD matrix formula, as shown in Fig. S1a. In this cavity design, a laser beam starting from one of the flat end mirrors situated at the origin in the simulation was first reflected from an Yb:YAG thin disk (TD) placed approximately 10 m away from the end mirror. The radius of curvature of the Yb:YAG TD was assumed to be 4 m by considering the thermal deformation of the TD. The laser beam should be reflected one more time with the same Yb:YAG TD to acquire a sufficient laser gain, while the beam diverges with the first reflection from the concave surface of the Yb:YAG TD. Therefore, we put a concave mirror with a curvature of 5 m at the center of two reflections from the Yb:YAG TD to configure a telescope with the three reflections from the Yb:YAG TD, concave mirror, and again Yb:YAG TD. We henceforth call this concave mirror ‘thermal lens compensation concave mirror (TLCCM)’ for simplicity. After approximately 20 m propagation, the laser beam passed through a telescope consisting of a pair of concave mirrors with a curvature of 5 m to stabilize the oscillation condition. Behind this telescope, another telescope consisting of a pair of concave mirrors with a curvature of 0.4 m tightly focused the laser beam to replicate a HHG port. The pair of convex mirrors with a curvature of −5 m sandwiched the tightly focusing telescope to reduce the focal spot diameter by increasing the beam diameters at the concave mirrors. Finally, the laser beam reached the flat end mirror placed on the other side of the cavity. This is a basic configuration to demonstrate continuous wave (CW) laser oscillation in the linear cavity. The resultant evolution of the beam radius of the CW laser oscillation in the cavity is shown as a solid red curve in Fig. S1a.

To mimic Kerr lens modelocking, we put a lens with a focal length of 100 m between the TLCCM and the second reflection from the Yb:YAG TD. The focal length of the lens is similar to that of a Kerr lens estimated from the intensity of a mode-locked pulse in the cavity and the nonlinear refractive index of a YAG plate. By inserting the Kerr lens, the beam shows a decrease in radius at almost the entire region in the cavity compared with the CW laser oscillation, shown as a blue solid curve in Fig. S1a Hence, we put a hard aperture at the position of approximately 34 m from the first end mirror to induce a loss to the CW laser oscillation in the experiment.

We also found the difference in the stability range of the cavity between the CW laser oscillation and the Kerr lens mode-locking oscillation, as shown in Fig. S1b. We adjusted the position of the TLCCM between the two reflections from the Yb:YAG TD in this calculation. The adjusting parameter denoted as Δ*L* in Fig. S1b is defined as a deviation from the position where the stable condition of the CW oscillation starts (6.973 m away from the first reflection of the Yb:YAG TD). We clearly observed that the stable region for the mode-locking laser oscillation was shifted approximately −20 mm from that for the CW oscillation. On the basis of this analysis of stability conditions, we fixed two pairs of flat mirrors with an incident angle of 45 degrees (four mirrors in total) onto a linear translation stage such that the TD-TLCCM and TLCCM-TD distances could be scanned equally and simultaneously in the entire range of stable conditions with an accuracy of ~μm in the actual setup of the linear cavity. The performance of the linear cavity is summarized in Figs. S2a, S2b, S2c, and S2d. Before starting modelocking, we adjusted the directions of the end mirrors and the position of the TLCCM to maximize the output power from one of the end mirrors (5 % output coupler), then we carefully moved the translation stage for the stable region for modelocking and vibrated it to start the modelocking. The measured output power from the mode-locked oscillator is shown in Fig. S2a. The equivalent intracavity power is indicated on the right axis of this figure. We successfully increased the intracavity power up to 2980 W at the pumping power of 1600 W under the stable mode-locking condition. We observed the pulse train with a PIN photodiode and concluded that the repetition rate of the mode-locked pulse was 2.85 MHz and there was neither Q-switching nor subpulses between the mode-locked pulses with an interval of 351 ns. Therefore, the pulse energy of the mode-locked pulse inside the cavity was estimated to be 1.04 mJ, which was the highest pulse energy, to the best of our knowledge, ever obtained in the mode-locked oscillator cavity. The autocorrelation traces of the output pulses are shown in Fig. S2b. We observed that the widths of the autocorrelation traces decreased with increasing the output power. This was consistent with the increase in spectral width, as shown in Fig. S2c, which was due to the fact that the spectral broadening with the self-phase modulation (SPM) in a plate of YAG (Kerr medium) was enhanced with the increase in the intensity of an intracavity pulse. The evolution of pulse duration with the change in pulse energy is shown as solid circles in Fig. S2d. The pulse durations in this figure were estimated from fits of the measured autocorrelation traces to the autocorrelation traces calculated by assuming the sech^2^-shaped temporal profile. The evolution of the spectral width is also shown as squares in the same figure. As a result, we obtained the shortest pulse duration of 520 fs at the maximum pulse energy of 1.04 mJ, and thus, the peak power was 1.8 GW. The peak intensity at the focal point of the mimicking HHG port was estimated to be 3.3×10^14^ W cm^-2^, which was sufficiently high to realize HHG.

**B. Design of ring cavity**

We designed the ring cavity on the basis of the design of the above-mentioned linear cavity successfully operated as a mode-locked oscillator. This was performed by unfolding the linear cavity with respect to the second end mirror. Namely, we removed the second end mirror of the linear cavity, put the mirror image of the sequence optics shown in Fig. S1a, and imposed the periodic boundary condition at both ends of the cavity. In the mirror image of the sequence of optics, we replaced the two reflections of the Yb:YAG TD to two concave mirrors with curvatures of both 5 m. The curvature of the TLCCM in the mirror image was also changed to 5 m. The second HHG port in the mirror image was shifted near the first HHG port. We configured a pair of YAG plates symmetrically to the TLCCM so that the total focusing power of the Kerr lenses from these YAG plates was similar to that from the YAG plate in one round trip of the linear cavity.

The unidirectional propagation of the mode-locked pulses in the ring cavity was crucial for HHG. It was known that this could be realized by introducing the asymmetry of the cavity to the Kerr lens effect [32], and thus we put an aperture only between the the Yb:YAG TD and the YAG plate situated at only one side of the TLCCM.

The resultant optics configuration and the beam radii calculated in the forward and backward directions are shown in Fig. S3. We arrange the main part of the mirror image of the optics sequence in the linear cavity on the left-hand side of the Yb:YAG TD. The forward direction is defined as a left-to-right direction and the aperture is put on the left-hand side of the TLCCM. The aperture is treated as a Gaussian spatial filter so that it can be represented by an ABCD matrix. The Kerr lens effect of a Brewster plate in the second HHG port was included in this calculation, whereas that in the first HHG port was excluded for simplicity

We found that the radius of the beam propagating backward in the cavity, shown as a blue solid curve in Fig. S3, was significantly larger than that of the beam propagating forward, shown as a red solid curve in Fig. S3, in the region between the Yb:YAG TD and the TLCCM except for the region near the focusing points. This asymmetry of radius evolutions in the forward and backward directions appeared even though the beam diameter suddenly decreased at the Gaussian spatial filter in the backward direction, as shown in the enlarged view of the blue solid curve. We confirmed that this situation was reversed by placing the aperture on the right-hand side of the TLCCM. The result suggests that a significant loss for the backward propagation suppressed the mode-locked oscillation in this direction; hence, the unidirectional mode-locked oscillation should be realized. On the basis of this design and analysis, we built a ring cavity with a length of approximately 100 m. Modelocking was achieved by carefully adjusting the position of the TLCCM and vibrating it, after optimizing the cavity condition so as to obtain the maximum output power of the CW laser oscillation. The performance of modelocking is described in the main text.

**ESTIMATION OF HH PHOTON FLUX**

The HH pulses generated at the tight focusing point in the first HHG port of the mode-locked oscillator propagated coaxially with the driving fundamental laser pulse and were reflected by the sapphire Brewster plate in the first HHG port. Then, the HH pulses passed through an aperture [not shown in Fig. 1a] and were focused by a gold-coated toroidal mirror into the entrance slit of an XUV spectrograph connected to the first HHG port, as described in the main text. There was no spatial clipping during this HH pulse transport owing to the nature of the HH pulse with small divergence. Using this setup, we recorded the HH spectra generated from Ne, Ar, and Xe gas targets, as shown in Figs. 3a-3c, respectively.

The XUV spectrograph consisted of an entrance slit, an aluminium filter, a toroidal-aberration-corrected grating working in the grazing incidence, and a back-illuminated X-ray CCD camera as a detector. Therefore, we converted the recorded spectral profiles to photon flux density (the number of photons / unit time / unit photon energy) at the HHG source in the cavity by taking into account (i) the quantum efficiency of the X-ray CCD camera, (ii) the diffraction efficiency of the toroidal grating, (iii) the transmittance of the aluminium filter, (iv) the throughput of the entrance slit, (v) the reflectivity of the toroidal mirror, and (vi) the reflectivity of the Brewster plate. We show the spectral profiles of five of these six quantities [(i), (ii), (iii), (v), and (vi)] in Fig. S4. The throughput of the entrance slit, (iv), was estimated to be 19 %. The resultant spectral profiles of the photon flux densities from the Ne, Ar, and Xe gas targets are shown in Figs. S5a, S5b, and S5c, respectively. The Ne, Ar, and Xe gas flow rates were adjusted to 570 ml min.^-1^, 5.3 ml min.^-1^., and 0.14 ml min.^-1^, respectively, in these measurements. A photon flux of one harmonic component was obtained by integrating the spectral profile of the photon flux density only around the peak photon energy of the harmonic component.

**GAS FLOW DEPENDENCE OF INTRACAVITY HHG**

We searched for the optimum HH yield by changing the flow rate of the Ar gas target in the first HHG port. The measured HH yield at each order with scanning gas flow ($\rho_{\mathrm{Ar}}$) is shown in Fig. S6 a. The HH yield is converted to absolute photon flux ($n_{\mathrm{ph}}$) by taking into account the efficiencies of the optics and apparatuses to observe the HH spectrum. The photon fluxes of H25 and lower harmonic orders exponentially increased in accordance with the increase in gas flow rate from 4 to 12 ml min.^-1^ and with a power law of $n_{\mathrm{ph}}\propto\rho_{\mathrm{Ar}}^{m}$, where the exponent *m* for the H25 and lower harmonic orders are all approximately estimated to be 2.2. The slow increase in the $n_{\mathrm{ph}}$ of all harmonic orders was observed with further increase in $\rho_{\mathrm{Ar}}$. The maximum photon flux of H17 described in the main text was obtained at a gas flow rate of 20 ml min.^-1^.

Note that there was no need for us to adjust the laser cavity condition with the increase in gas flow rate from 0 to 12 ml min.^-1^, whereas a change-by-change adjustment of the cavity was required to keep the modelocking stable for a gradual increase in gas flow rate from 12 to 20 ml min.^-1^. We were unable to achieve modelocking at a gas flow rate of more than 20 ml min.^-1^. The increase in nonlinear phase shift in the Ar gas target with the increase in gas flow rate should be compensated for by decreasing the nonlinear phase shift in a Kerr medium (a Brewster plate made of YAG crystal) to maintain the modelocking. Therefore, we assume that the nonlinear phase shift in the Ar gas target was negligibly small compared with that in the Kerr medium at gas flow rates lower than 12 ml min.^-1^. In the adjustment of the cavity at gas flow rates higher than 12 ml min.^-1^, we shifted the position of a concave mirror situated next to the Yb:YAG thin disk in the cavity with a motorized translation stage and altered the pumping laser diode power. The nonlinear phase shift in the Kerr medium was reduced so as to compensate for the additional nonlinear phase shift in the Ar gas target with this adjustment. We observed the incremental spectral narrowing and pulse broadening of the output pulse from the mode-locked oscillator induced by the increase in Ar gas flow rate depicted in Figs. S6b and S6c, as a proof of this supposition. The termination of modelocking at a gas flow rate higher than 20 ml min.^-1^ might be attributed to the low intensity of the laser pulse in the Kerr medium owing to the above-mentioned reason, or it might come from the instability of the high gas flow rate.

**LINEWIDTH OF HH**

We evaluated the bandwidths of harmonic pulses generated from three gas targets at each order. The result is shown in Fig. S7. The photon energy bandwidth ratio against the peak photon energy, Δ*E*/*E*, at each harmonic order is plotted in this figure. The bandwidth ratio is approximately independent of the kind of gas target used at the same harmonic order and monotonically increases from 3×10^-3^ at H13 for the Xe gas target to 8×10^-3^ at H43 for the Ne gas target with increments of the harmonic order. The increase in bandwidth is consistent with the evaluations of the coherence time of HH pulses reported in [S1]. The measured bandwidth at each harmonic order is sufficiently small for the application of high-resolution photoemission spectroscopy combined with femtosecond time-resolved measurement [S2].

**References**

[S1] Lyngå, C. et al. Temporal coherence of high-order harmonics. *Phys. Rev. A* **60**, 4823–4830 (1999).

[S2] Yoshida, R. et al. Ultrafast photoinduced transition of an insulating VO thin film into a nonrutile metallic state. *Phys. Rev. B* **89**, 205114 (2014).


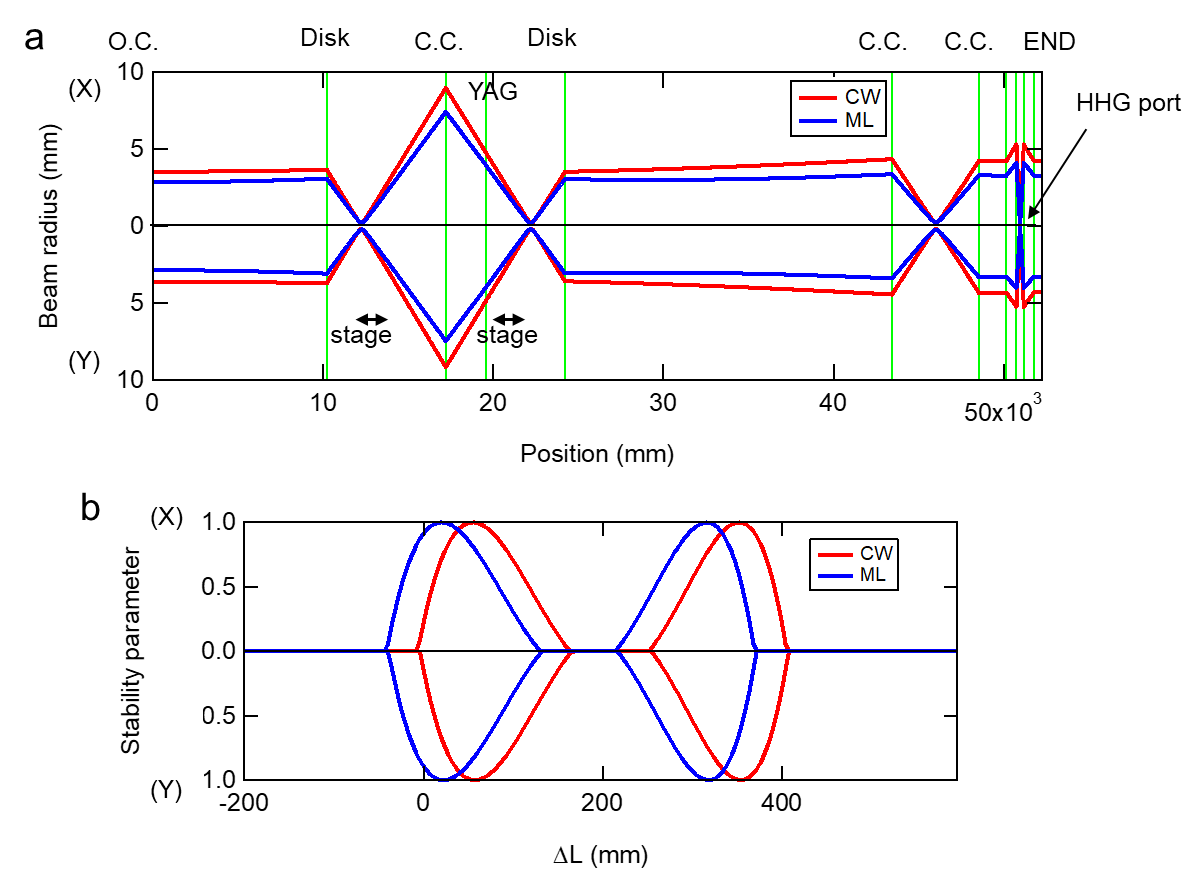


Fig. S1. (a) Design of linear cavity. We indicate the positions of optics with yellow—green lines under the names of optics. The radius of the beam in the cavity without considering the Kerr lens in a YAG plate is depicted as a solid red curve (CW) and that including the Kerr lens at the YAG position is depicted as a solid blue curve (ML). C.C.: concave mirror. Disk: Yb:YAG TD acting as a concave mirror. YAG: YAG plate as a Kerr medium. O.C.: output coupler (first end mirror). END: (second) end mirror. (b) Stability parameters with scanning position (Δ*L*) of thermal lens compensation concave mirror (TLCCM) relative to Yb:YAG TD with (ML, blue solid curve) and without (CW, red solid curve) Kerr lens, respectively. The TLCCM is indicated as ‘C. C.’ between the two ‘Disks’ in Fig. S1a. The origin of Δ*L* is defined as the position where the stable range starts for the CW oscillation. In the actual calculation, the distance from the ‘Disk’ on the left-hand side to the ‘C.C.’ and that from the ‘C.C.’ to the ‘Disk’ on the right-hand side are simultaneously altered by Δ*L*.


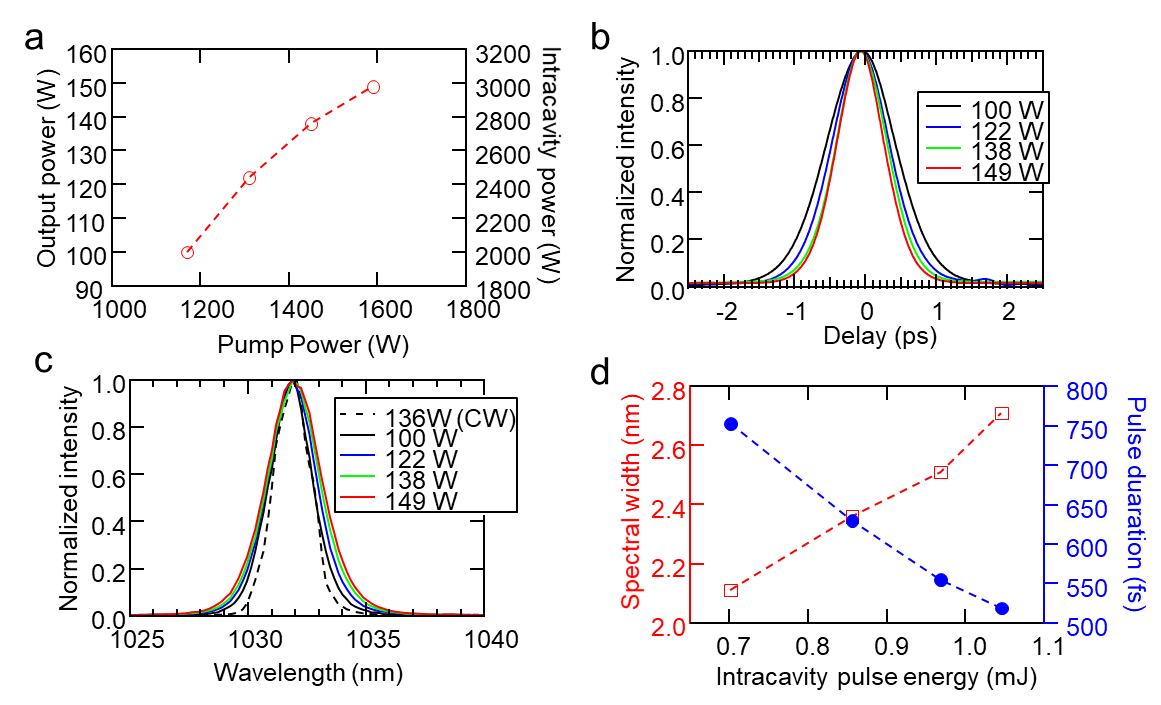


Fig. S2. (a) Output power of mode-locked oscillator with 50 m linear cavity. The intracavity power estimated from the output power is indicated on the right axis. (b) Autocorrelation traces of output pulses at output powers of 100 W (black solid curve), 122 W (blue solid curve), 138 W (yellow-green solid curve), and 149 W (magenta). (c) Spectra of the output pulses at output powers of 100 W (black solid curve), 122 W (blue solid curve), 138 W (yellow-green solid curve), and 149 W (magenta). The spectrum of the CW output at an output power of 136 W is also indicated as a dashed black curve. (d) Evolutions of spectral width and pulse duration to intracavity pulse energy.


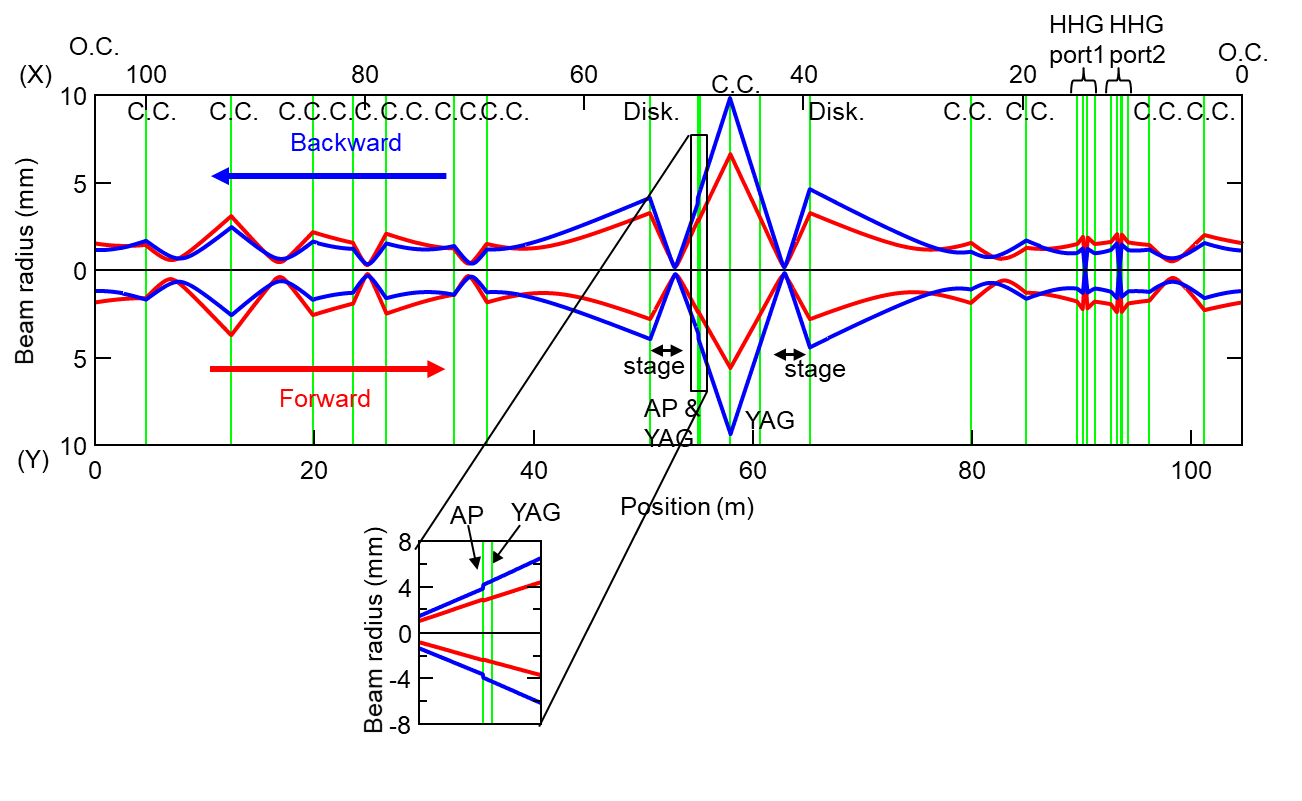


Fig. S3. Design of ring cavity. We indicate the positions of optics with yellow-green lines under the names of optics. The radius of the beam propagating forward (left to right) is depicted as a solid red curve and that of the beam propagating backward (right to left) is depicted as a solid blue curve. An enlarged view of these curves near the aperture is shown as an inset. C.C.: concave mirror. Disk: Yb:YAG TD acting as a concave mirror. YAG: YAG plate as a Kerr medium. AP: aperture. HHG port 1 consists of a pair of tightly focusing concave mirrors set between a pair of convex mirrors to increase the beam radius on each concave mirror. Configuration of HHG port 2 is the same as that of HHG port 1.


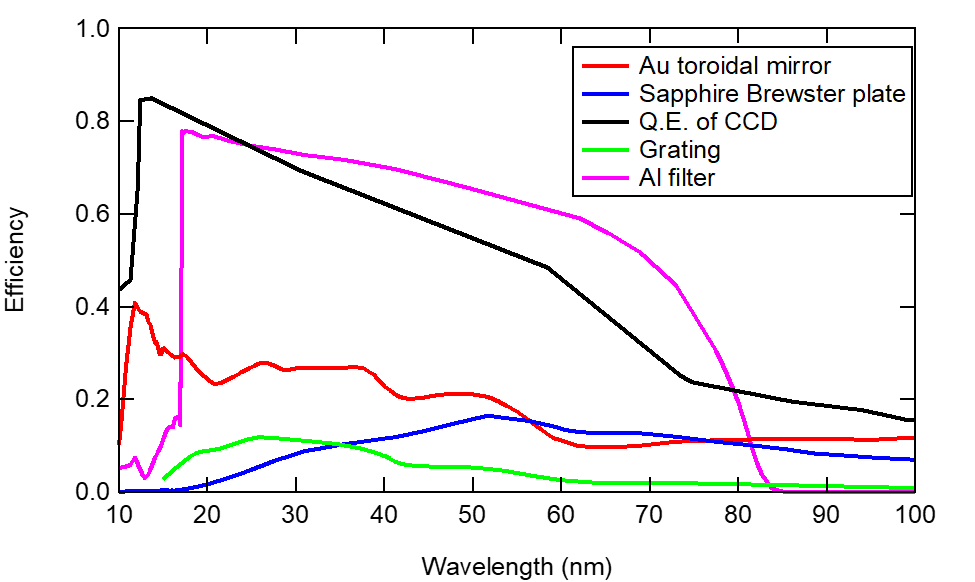


Fig. S4. Spectral responses of detector and optics used for measuring spectral profiles of HH pulses. Red solid curve: reflectivity of toroidal mirror. Blue solid curve: reflectivity of Brewster plate. Black solid curve: quantum efficiency of X-ray CCD camera. Green solid curve: diffraction efficiency of toroidal grating. Magenta solid curve: transmittance of aluminum filter.


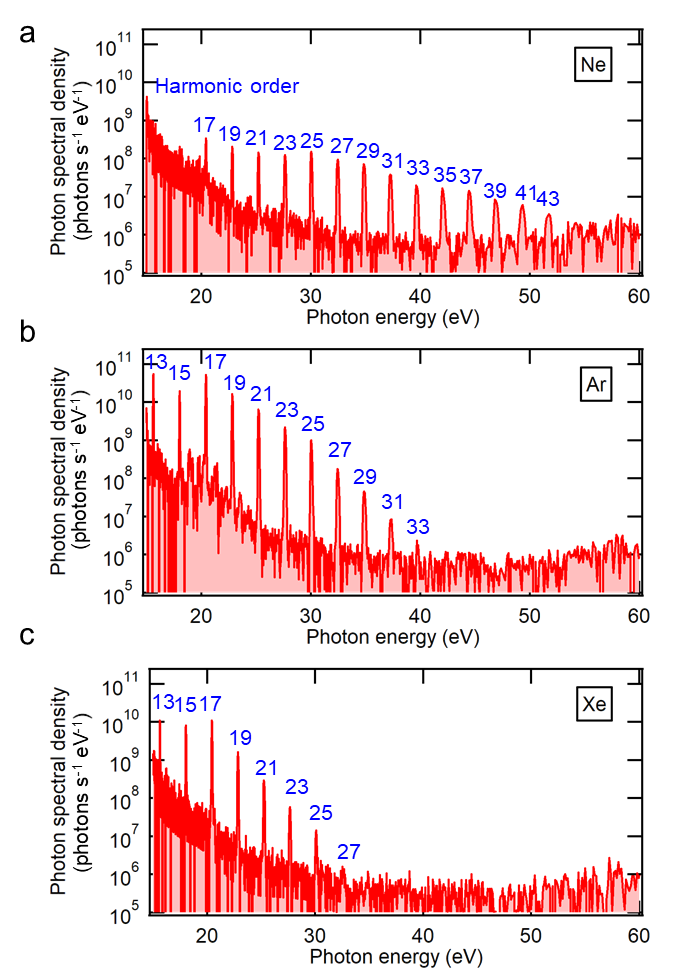


Fig. S5. Spectral profiles of photon number densities of HH generated from (a) Ne, (b) Ar, and (c) Xe. These spectra were independently acquired under the single-HHG-port operation of the mode-locked laser.


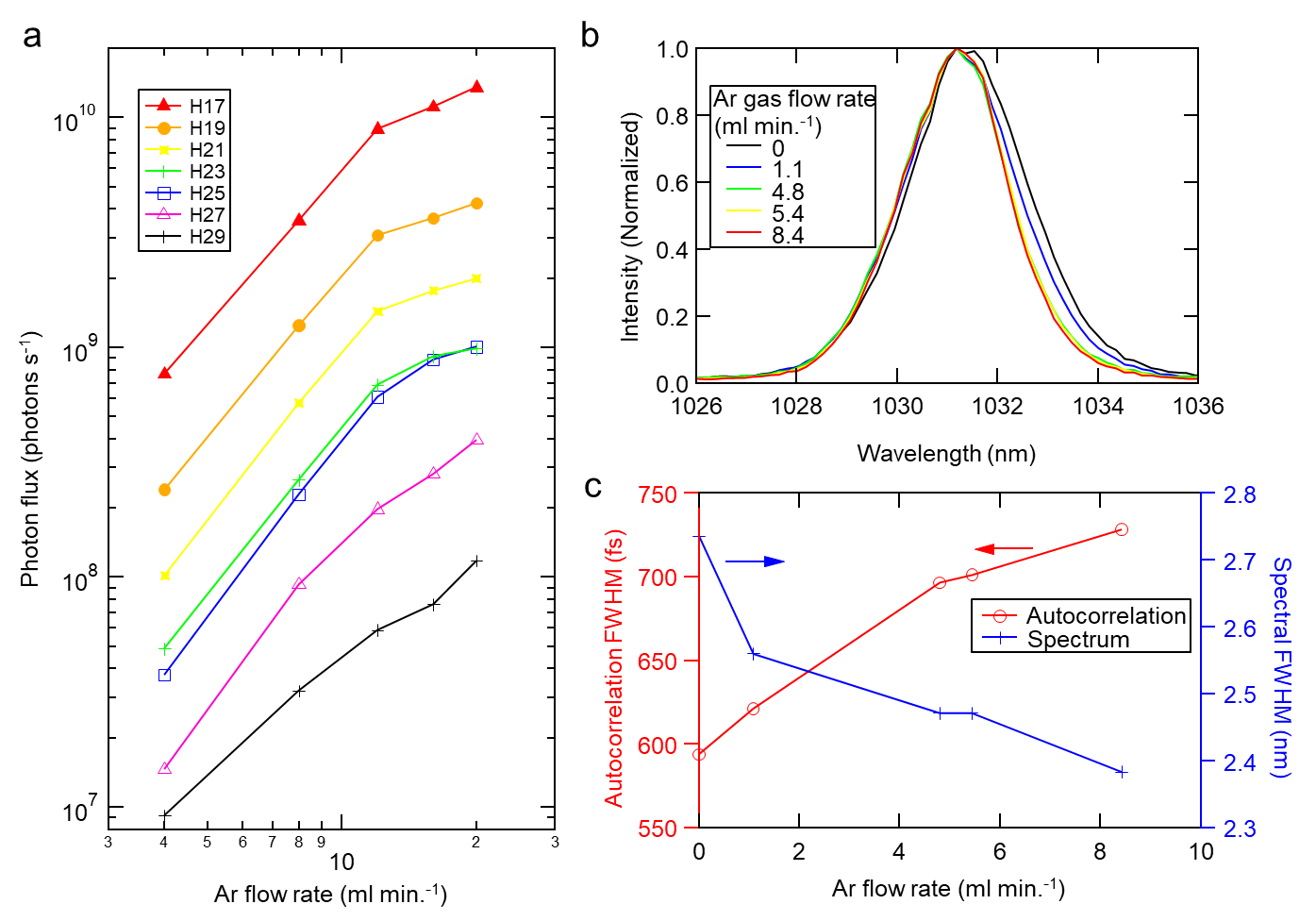


Fig. S6. (a) Evolution of photon flux of each HH component with increase in Ar gas flow rate in first HHG port. (b) Spectral profiles of output pulse from mode-locked oscillator at different gas flow rate of Ar gas in first HHG port. (c) Evolution of spectral (crosses) and pulse (circles) durations of output pulse from mode-locked oscillator with increase in flow rate of Ar gas in first HHG port.


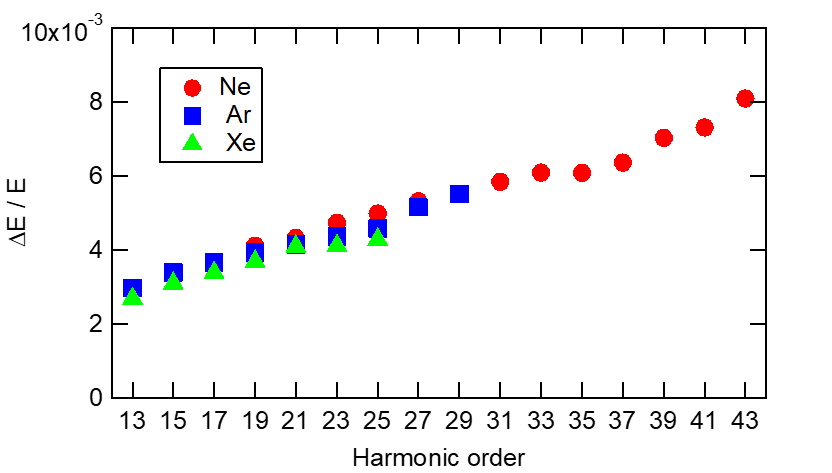


Fig. S7. Photon energy bandwidth (Δ*E*) ratio against peak photon energy (E) at each HH component generated from Ne (circles), Ar (squares), and Xe (triagles) gas targets.
